# Chiral Recognition Mechanism of Amlodipine by Capillary Electrophoresis Using Carboxymethyl-β-cyclodextrin: An Experimental and Theoretical Study

**DOI:** 10.1021/acsomega.4c09559

**Published:** 2025-01-21

**Authors:** Camilla Fonseca Silva, Clebio Soares Nascimento, Keyller Bastos Borges

**Affiliations:** † Department of Chemistry, ICEX, Federal University of Minas Gerais, Av. Presidente Antônio Carlos, 6627, Belo Horizonte, Minas Gerais 31270-901, Brazil; ‡ Department of Natural Sciences, DCNAT, Federal University of São João del-Rei, Campus Dom Bosco, Praça Dom Helvécio 74, Fábricas, São João del-Rei, Minas Gerais 36301-160, Brazil

## Abstract

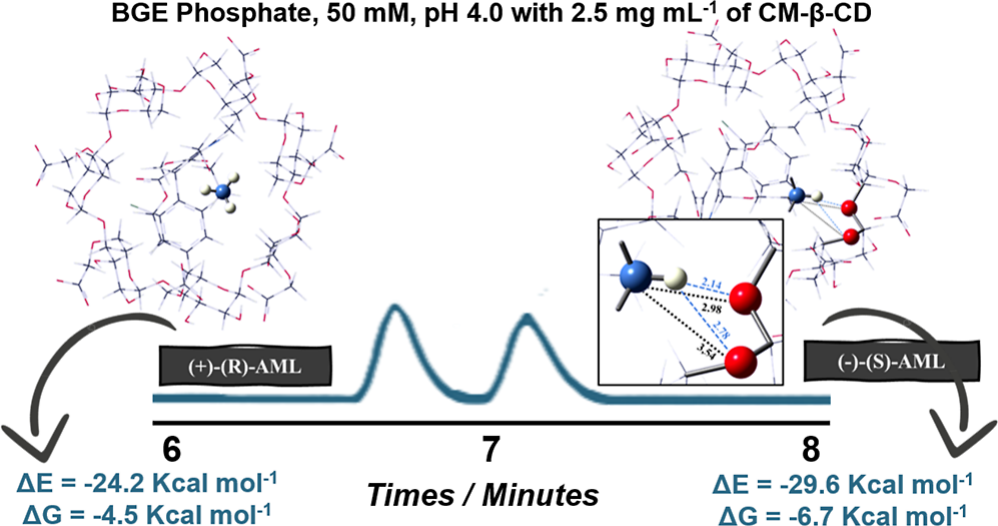

Chiral capillary electrophoresis (CCE) represents an effective technique for enantioselective separations. However, additional techniques may be necessary to determine the enantiomers migration order (EMO) and elucidate the chiral recognition mechanism. This study details the development and optimization of a CCE method for the enantioseparation of amlodipine (AML). Furthermore, it contributes computationally to determining the EMO and the mechanisms responsible for chiral discrimination. The study proposed the optimization of several key parameters in CCE, including the type, concentration and pH of the background electrolyte, as well as the concentration of the chiral selector. In line with previous research, only one anionic cyclodextrin, carboxymethyl-β-cyclodextrin (CM-β-CD), was evaluated as the chiral selector. Following optimization, the most favorable results were achieved using 50 mM phosphate background electrolyte pH 4.0 with 2.5 mg mL^–1^ CM-β-CD. These conditions enabled baseline separation of AML enantiomers, reduced analysis time, and minimized consumption of the chiral selector. Calculations were conducted using a sequential methodology, beginning with the PM3 semiempirical followed by density functional theory (DFT) calculations. The theoretical analysis indicated that differences in Δ*E* and Δ*G* values are reliable indicators for predicting the EMO. Specifically, the (−)-(*S*)-AML/CM-β-CD complex exhibited superior energetic characteristics compared to the (+)-(*R*)-AML/CM-β-CD complex, likely due to differences in their intermolecular interactions, including hydrogen bonds and electrostatic interactions, consequently, this finding can be related to elongation migration time within the electrophoretic system. These results underscore the synergistic benefits of computational and experimental approaches in elucidating chiral discrimination mechanisms and identifying EMO in CCE.

## 1 Introduction

**Figure 1:**
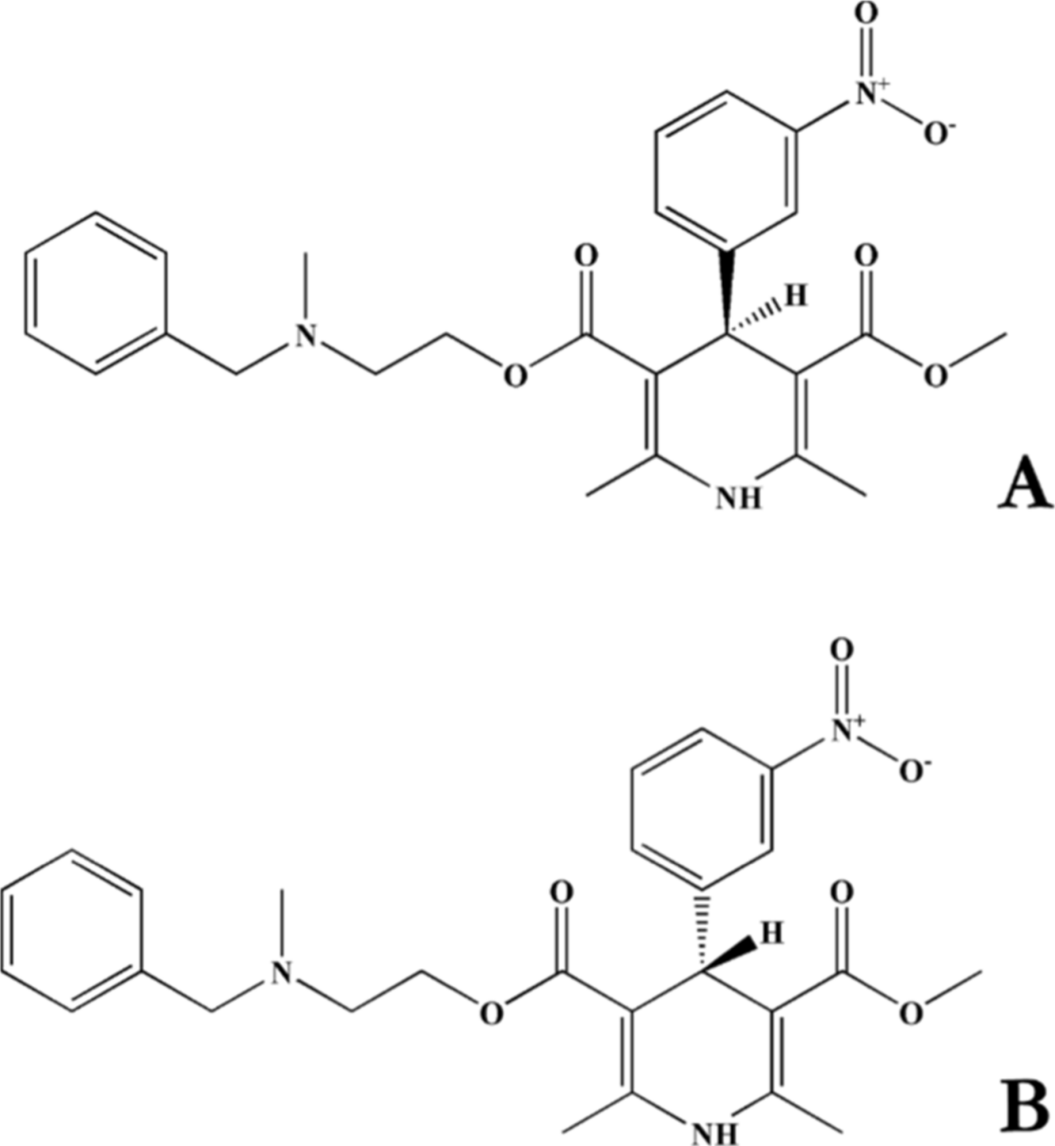
Structures of enantiomers: (a) (−)-(*S*)-AML and (b) (+)-(*R*)-AML.

Amlodipine (AML) (Figure 1), a calcium channel blocker (CCB) with antihypertensive and antianginal properties, is administered therapeutically as a racemic mixture.^1−3^ Nonetheless, previous investigations have established that the (−)-(*S*)-AML enantiomer is primarily responsible for its pharmacological efficacy.^4,5^ Considering the disparities in the pharmacokinetic behavior of enantiomers, enantioselective analytical methodologies have gained prominence in scientific literature in recent years. One such technique is chiral capillary electrophoresis (CCE), which offers several advantages in terms of analysis time, efficiency, and simplicity.^6−8^

Enantioseparations using CCE are achieved by incorporating chiral selectors into the background electrolyte (BGE). These chiral selectors form distinct diastereomeric complexes with the enantiomers, analogous to a host–guest system. The distinct spatial arrangements of the enantiomers lead to varying affinities for the cavities within the chiral selectors. A variety of intermolecular forces contribute to the formation of these complexes, including commonly van der Waals, π–π stacking, and hydrogen bonding.^8^

Cyclodextrins (CDs), comprising approximately 80% of chiral selectors utilized in enantioseparation through CCE, represent the predominant option in comparison to other alternatives, including proteins, crown ethers, polysaccharides, macrocyclic glycopeptides, and cinchona alkaloids.^9−11^ The mechanism of chiral recognition by CD involves the formation of an inclusion or association complex with each enantiomer. Table 1 summarizes recent investigations into the enantioselective separation of AML via CCE employing CDs as chiral selectors.^12−24^

**Table 1:** Main Published Works Reporting BGE and EMO Conditions for the Determination of AML Enantiomers by CE with CD as a Chiral Selector^a^

BGE and CD	EMO	references
phosphate 100 mM (pH 2.0) + 5 mM HP-α-CD	R, S	(12)
acetonitrile: 20 mM NaH_2_PO_4_ (pH 3.95) + 20 mM SBE-β-CD	S, R	(13)
phosphate 50 mM (pH 3.0) + 20 mM RAMEB	R, S	(14)
glycine-acetate 50 mM (pH 3.2) + 50 mg mL^–1^ HP-β-CD		(15)
Tris-phosphate 30 mM (pH 3.0) + α-CD 3.0% (w/v)	R, S	(16)
glycine-acetate 50–168 mM (pH 3.2) + 50 mg mL^–1^ HP-α-CD		(17)
phosphate 25 mM (pH 9.0) + 15 mM CM-β-CD	S, R	(18)
phosphate 100 mM (pH 3.0) + 5 mM HP-α-CD	R, S	(19)
phosphate 40 mM (pH 6.1) + 5 mM EDA-β-CD		(20)
phosphate 30 mM (pH 3.0) + 0.0645 mM CM-β-CD		(21)
phosphate 50 mM (pH 3.0) + 18 mM α-CD		(22)
phosphate 75 mM (pH 2.5) + 15 mM HP-α-CD	R, S	(23)
acetate 20 mM (5.0) + MDI-β-CD column	R, S	(24)

a HP-α-CD: hydroxypropyl-α-CD, SBE-β-CD: sulfobutylether-β-CD; RAMEB: randomly methylated-β-CD; CM-β-CD: carboxymethyl-β-CD; EDA-β-CD: ethanediamine-β-CD; MDI-β-CD: diisocyanate-β-CD-modified.

It is worth noting that although the use of different BGEs and CDs has been reported, some special care in choosing the conditions employed invariably leads to better results in terms of resolution (*R*_s_) and analysis time. For example, when using the conventional polarity mode, with detection at the cathode (−), the positive charge on the species to be determined favors better *R*_s_ and shorter migration time (*T*_m_). Additionally, in CCE, the use of modified and charged CDs, with the opposite charge to the analyte to favor their interactions, offers advantages over other available CDs, such as natural β-CDs.

Theoretically, for the separation of AML enantiomers (p*K*_a_ = 9.28), the use of BGEs with pHs lower than 7.0 containing negatively charged CDs, such as carboxylated (–COO^–^) and sulfated (–SO_3_^–^), favors their species being 100% cationic, therefore with electrophoretic mobility toward detection and with the opposite charge to the selectors used, forming specific electrostatic interactions, which in turn increase separation efficiency, as reported by Owens and Gong.^13,21^ According to the systematic study of the influence of neutral and anionic CDs on AML separation, Owens et al. defined that anionic CDs as CM-β-CD, offered improved separation compared to neutral CDs and that lower concentrations were required to achieve good *R*_s_ and lower *T*_m_.

Given the diversity of conditions to be used for separation by CCE, comprehending the nature of the forces governing the affinity pattern of each enantiomer can offer valuable insights into the enantiomer migration order (EMO). It is worth highlighting that the studies lack detailed insights into the molecular structures of the diastereomeric complexes formed and the EMO, representing a significant gap in current research. To address this gap and advance our understanding, further research is imperative in this domain.^25^ Traditional methods for determining EMO rely on the analysis of pure enantiomers using techniques like circular dichroism, polarimetry, or NMR in conjunction with X-ray crystallography.^26^ However, these approaches are often hindered by high costs and limited commercial availability of enantiomer standards. To circumvent these limitations, computational studies on diastereomeric complexes have emerged as a viable alternative. These theoretical investigations offer a reliable means of predicting EMO by scrutinizing the structural, topological, electronic and energetic properties of the diastereomeric complexes.^27^

A comprehensive literature review published in 2021^28^ synthesized a variety of studies conducted between 2016 and 2021, which employed computational and experimental methods to investigate the mechanisms underlying chiral discrimination in CCE using CD. Numerous research groups have focused on elucidating these mechanisms using diverse theoretical techniques. These approaches have included classical molecular dynamics and molecular docking simulations, as well as hybrid quantum mechanics/molecular mechanics (QM/MM) methods, semiempirical methods (SEM), and purely quantum-based density functional theory (DFT) calculations.

In this context, the present study sought to identify the optimal conditions for the enantioselective separation of AML, with a particular focus on the influence of key electrophoretic parameters, as nature, concentration, and pH of the BGE, as well as the concentration of CM-β-CD. In this context, the study aimed to determine the optimal conditions for the enantioselective separation of AML, emphasizing the role of key electrophoretic parameters, including the nature, concentration, and pH of the BGE, as well as the concentration of CM-β-CD. The selection of CM-β-CD as an anionic chiral selector was guided by its demonstrated ability to form stable diastereomeric complexes with cationic analytes, as evidenced in numerous studies.^29−33^ Its proven versatility and effectiveness across diverse analyte classes further reinforce its reliability in advanced separation techniques.

After the completion of the experimental analysis, theoretical calculations were conducted with the objective of elucidating the molecular structures and energetics of the diastereomeric complexes, thereby facilitating the determination of the EMO. By integrating experimental and theoretical methodologies, this study offers a comprehensive understanding of the mechanisms underlying chiral recognition through computational modeling. This integrative approach represents a significant advancement compared to previous studies on AML enantioselective analysis, which have predominantly relied on experimental techniques.

## 2 Materials and Methods

### 2.1 Chemicals and Standard Solutions

AML standard solutions (stock) were prepared in HPLC grade methanol purchased from Dinâmica (Diadema, SP, Brazil) at a concentration of 0.1 mg mL^–1^ and stored at −20 °C in the dark. For CE analyses, stock solutions were diluted to a final concentration of 0.01 mg/mL by mixing in a 1:1:8 (v/v/v) ratio of AML, BGE, and ultrapure water. The anhydrous monobasic sodium phosphate salts (NaH_2_PO_4_, 98%) (Neon, São Paulo, SP, Brazil), sodium citrate dihydrate (NaH_2_PO_4_, 98%), sodium acetate sodium dihydrate (NaH_2_PO_4_, 98%), phosphoric acid dihydrate (NaH_2_PO_4_, 98%) and sodium hydroxide (NaOH), all obtained from Synth, São Paulo, SP, Brazil were used to prepare the BGEs.

The BGEs were prepared on the day by weighing the appropriate quantities of salts for each concentration: 0.153 g (25 mmol L^–1^ NaH_2_PO_4_), 0.306 g (50 mmol L^–1^ NaH_2_PO_4_), 0.459 g (75 mmol L^–1^ NaH_2_PO_4_), 0.612 g (100 mmol L^–1^ NaH_2_PO_4_) 0.750 g (50 mmol L^–1^ Na_3_C_6_H_5_O_7_) and 0.900 g (150 mmol L^–1^), with subsequent solubilization in 50 mL of ultrapure water, filtration through 0.45 μm Millipore Millex-GV PVDF hydrophilic filters and sonication for 10 min. The pH was adjusted using 0.1 and 0.01 mol L^–1^ NaOH and H_3_PO_4_ solutions.

The carboxymethyl-β-cyclodextrin sodium salt (CM-β-CD, catalog ID: 21906, mean degree of substitution: DS ∼ 3) was used as the CD during the study and obtained from Cyclolab (Budapest, Hungary). BGE solutions with chiral selector were also prepared daily using the following amounts of CM-β-CD for each concentration: 2.5 mg (1.625 mmol L^–1^ CM-β-CD), 4.0 mg (2.600 mmol L^–1^ CM-β-CD), 5.5 mg (3.575 mmol L^–1^ CM-β-CD), 7.0 mg (4.550 mmol L^–1^ CM-β-CD) and 8.5 mg (5.525 mmol L^–1^ CM-β-CD), with subsequent solubilization in 1.0 mL of BGE, filtration on 0.45 μm Millipore Millex-GV PVDF hydrophilic filters and sonication for 10 min. Ultrapure water was obtained using the Millipore Milli-Q Plus system (Bedford, MA, USA).

### 2.2 Instrumentation and Electrophoretic Conditions

Capillary electrophoresis equipment model CE7100 from Agilent Technologies was used to separate AML enantiomers. The equipment consisted of an analyzer, an automatic sampler and a diode array detector (DAD) operating at 195 nm. A capillary tube made of fused silica with a polyimide coating, measuring 75 μm in internal diameter, 71 cm total length, and 62.5 cm in effective length, was utilized. The tube was conditioned/washed before, during and after use according to the following protocol: (i) prior to first use, it was conditioned with a NaOH solution (1.0 M) for 10 min and then washed for another 30 min with ultrapure water; (ii) at the beginning and end of each day of use, conditioned for 10 min with the NaOH solution (0.1 M) and then washed for another 10 min with ultrapure water and (iii) between analyses, conditioned for 2 min with the NaOH solution (0.1 M), followed by washing for another 2 min with ultrapure water and finally, conditioning with the BGE + CD solution for 3 min. The hydrodynamic injection mode was used with pressure and time values of 30 mbar and 4 s, respectively and a separation voltage of 15 kV. The study assessed the impact of the chemical composition, concentration and pH of BGE, as well as the concentration of the CD.

### 2.3 Theoretical Methodology

Semiempirical PM3 and DFT calculations were conducted to elucidate the molecular mechanisms underlying the formation of diastereoisomeric complexes. These computational methods allowed us to investigate the structural and energetic properties related to this complexation process. In these calculations, AML appears as a cationic species (–NH_3_^+^) and CM-β-CD as anionic (–COO^–^) due to the deprotonated carboxyl groups at the chosen pH of 4.0, which aligns with the optimized condition determined from the experimental study.^14^

Despite the rapid increase in the number of articles involving DFT calculations in recent years, SEM continues to be of considerable interest due to its lower computational requirements. For instance, studies have consistently shown that SEM, including PM3 and PM6, can yield accurate predictions of molecular properties, particularly those related to molecular structure. Moreover, they offer significantly lower computational costs, making them an appealing choice characterizing diasteroisomeric complexes formed in CCE.^28^

The molecular geometries of CM-β-CD, (−)-(*S*)-AML, (+)-(*R*)-AML, as well as their respective complexes (−)-(*S*)-AML/CM-β-CD and (+)-(*R*)-AML/CM-β-CD (mode 1 and mode 2), were fully optimized in the gas phase without any geometrical or symmetry restrictions at the semiempirical PM3.^34^ To improve the reliability of the calculated energetic values, single-point calculations were then performed using DFT with the B97D functional [^35^-311+G(d,p) basis set,^36^ in the aqueous phase. The relative variation in electronic energy (ΔΔ*E*) and Gibbs energy (ΔΔ*G*) for the formation of the complexes was determined using eqs 1 and 2.
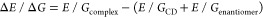
1
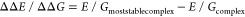
2

Solvation model based on density (SMD) was used to account for the solvent effect (water).^37^ In this model, the aqueous environment is represented by the dielectric constant of water (ε = 78.4). The solute molecule is placed within a cavity designed to encapsulate it entirely, which is then quickly encompassed by the continuum dielectric. All the calculations in this study were performed using Gaussian 09.^38^

## 3 Results and Discussion

### 3.1 Experimental Section

#### 3.1.1 Composition and Concentration of BGE

In capillary zone electrophoresis (CZE), the capillary is filled entirely with the same BGE consisting of charged particles (cations and anions). This facilitates the passage of electric current and maintains constant parameters such as conductivity, temperature, and pH throughout the analysis. When selecting the nature of the BGE, it is important to meet specific requirements related to its type and concentration. The BGE must provide a suitable medium for the migration of the analytes within a reasonable time frame, while also allowing for adequate detection in relation to the obtained peak profile. The first requirement to consider is the absorption of UV radiation by the BGEs constituent cations.

**Figure 2:**
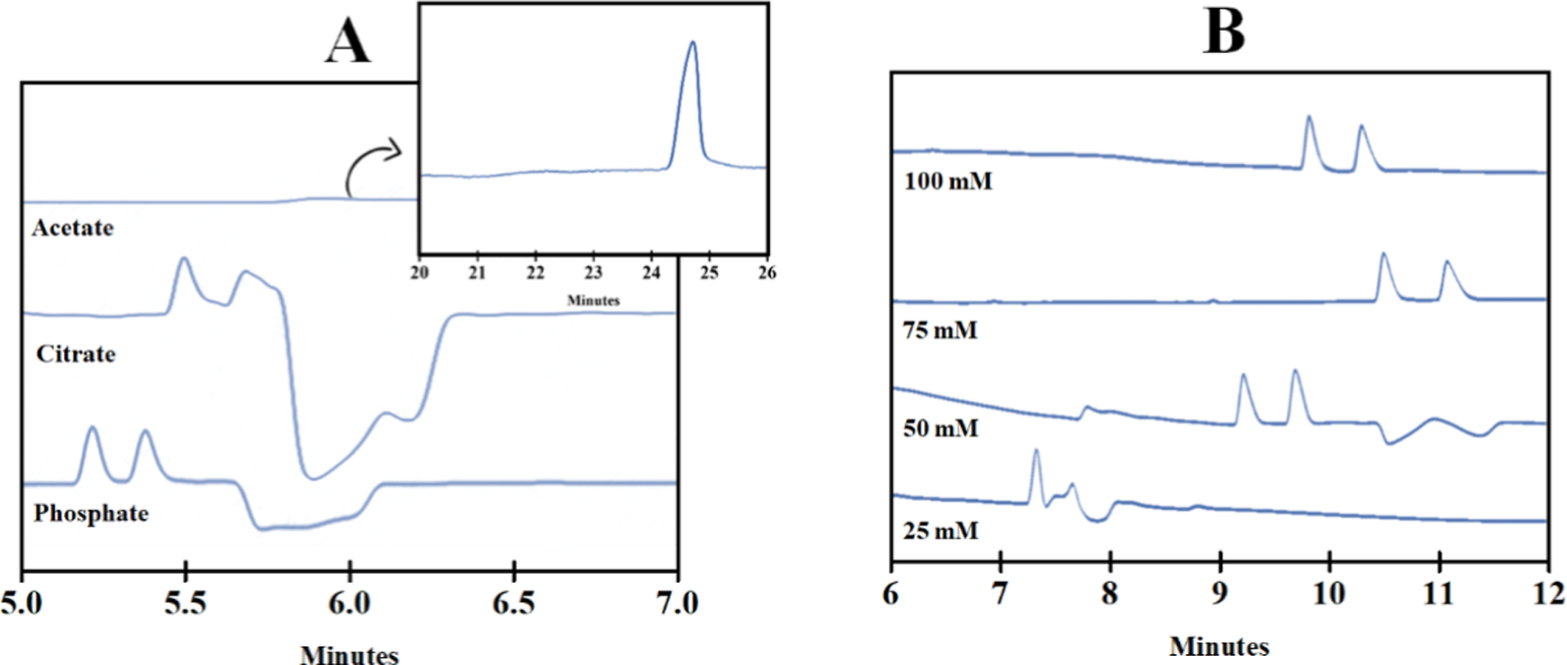
Electropherogram referring to (A) variation of BGE composition and (B) BGE concentration. Experimental conditions set in (A): 5.0 mg mL^–1^ CM-β-CD in 50 mM BGE pH 4.0 adjusted using 0.1 and 0.01 mol L^–1^ NaOH and H_3_PO_4_ solutions and in (B) 5.0 mg mL^–1^ CM-β-CD in BGE phosphate pH 4.0 adjusted using 0.1 and 0.01 mol L^–1^ NaOH and H_3_PO_4_ solutions. Uncoated fused-silica capillary 75 μm id × 62.5 cm (71 cm to the detector); hydrodynamic injection using 40 mbar × 4 s; separation voltage at 15 kV; UV detection at 195 nm.

The analysis of the AML analyte is conducted in direct UV mode due to its UV absorbance with a maximum of 195 nm. Therefore, the BGE ions used must be transparent to UV rays. It is recommended to use lithium, sodium, and potassium salts. For this reason, the BGEs evaluated in this study were prepared using species such as sodium phosphate, sodium citrate and sodium acetate, inevitably resulting in solutions with varying ionic strength (IS) values. As the IS of the solution depends on the charge of the ionic species (Zi) and the equilibrium concentration ([i]), the type of BGE significantly influences mobility and, consequently, selectivity. Figure 2A demonstrates that the enantiomers were only separated when using phosphate BGE and an appropriate current value (84 μA) was achieved.

Regarding the concentration of BGE, Figure 2B and Table 2, show that at a low concentration of BGE (25 mM), the electroosmotic flow (EOF) is also low, resulting in slower separation of enantiomers and decreased reproducibility of the method. At very high concentrations of BGE, increased current and heating of the medium (Joule effect) can affect separation. For the subsequent analyses, a 50 mM BGE phosphate was chosen based on the results obtained at this stage.

**Table 2:** Resolutions Found for Each Concentration of BGE Phosphate and CM-β-CD Evaluated^a^^,^^b^

concentration	resolution	current
BGE phosphate (mM)		
100	2.60	100
75	2.69	81
50	2.73	74
25	ncr	45
CD (mg mL^–1^)		
5.5	2.88	79
4.0	2.10	72
2.5	1.68	69

a ncr: no chiral resolution.

b Values of current in μA.

#### 3.1.2 pH of BGE

**Figure 3:**
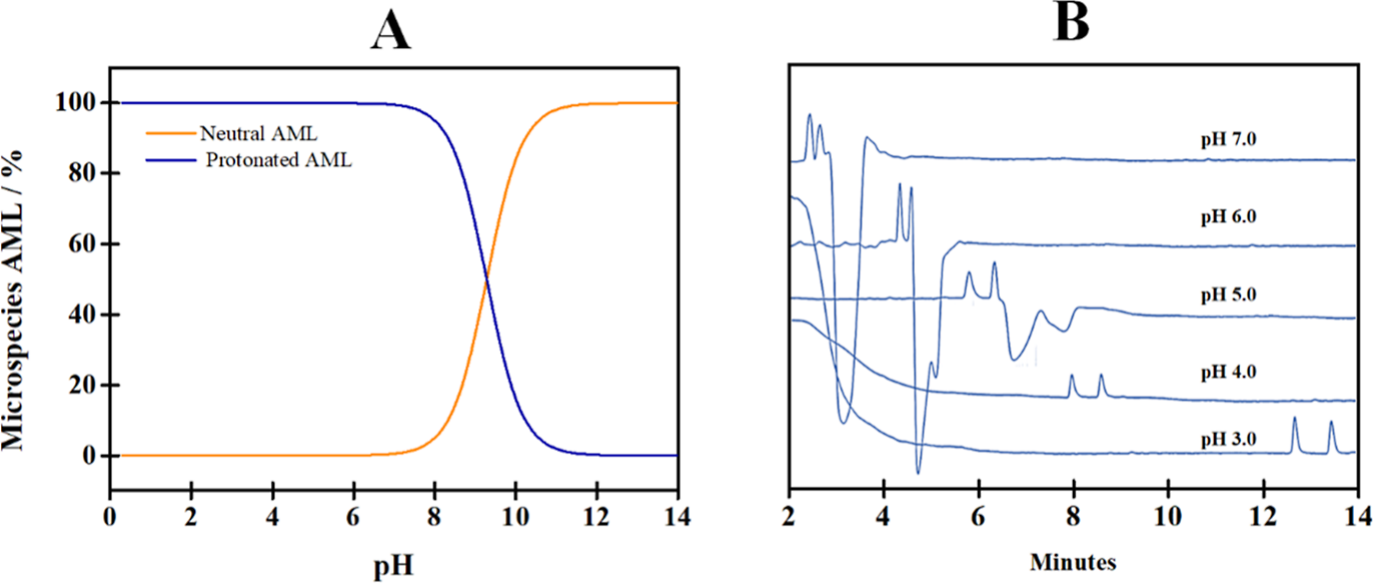
(A) Percentage of distribution of AML microspecies versus pH and (B) electropherogram referring to pH variation of BGE phosphate. Experimental conditions set in (B) 5.0 mg mL^–1^ CM-β-CD in 50 mM BGE phosphate pH 4.0 adjusted using 0.1 and 0.01 mol L^–1^ NaOH and H_3_PO_4_ solutions. Uncoated fused-silica capillary 75 μm id × 62.5 cm (71 cm to the detector); hydrodynamic injection using 40 mbar × 4 s; separation voltage at 15 kV; UV detection at 195 nm.

The impact of pH on CZE can be explained by examining the behavior of an uncoated fused-silica capillary. As established, at low pH values, the silanol groups become protonated (SiOH), leading to almost negligible EOF. On the other hand, at higher pH values, the silanols are completely deprotonated (SiO^–^), resulting in a high EOF. Additionally, adjusting the pH not only affects the intensity of the EOF but also the charge of the analyte, since AML has p*K*_a_ equal to 9.28. Thus, it is predicted that in BGEs with pH values lower than 9.28, the cationic microspecies of AML will predominate. Conversely, in pH conditions higher than 9.28, the neutral (or molecular) microspecies will predominate (Figure 3A).

Considering that AML injection occurs at the anode (positive polarity) and detection occurs at the cathode (negative polarity), AML must carry a positive charge to facilitate its detection through the mutual influence of its electrophoretic mobility and the EOF. Therefore, only acidic pH conditions were tested (ranging from 3 to 7), as shown in Figure 3B. It was observed that at higher pH values (5, 6 and 7), *T*_m_ were shorter and closer to the EOF signal, compromising the separation. Therefore, pH 4.0 was used instead of pH 3.0 due to the higher *R*_s_ values and shorter *T*_m_ values associated with it (see Table 3).

**Table 3:** Resolutions Obtained for Each pH of BGE Phosphate Evaluated^a^

pH	resolution	current
7.0	1.12	99
6.0	2.09	89
5.0	2.56	77
4.0	2.76	64
3.0	4.69	54

a Values of current in μA.

#### 3.1.3 Concentration of CD

**Figure 4:**
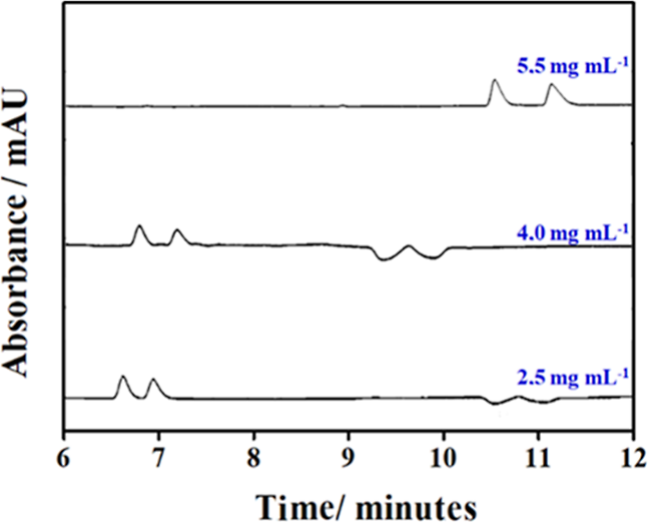
Electropherograms referring to variation in the concentration of CD. Experimental conditions: CM-β-CD in 50 mM BGE phosphate pH 4.0 adjusted using 0.1 and 0.01 mol L^–1^ NaOH and H_3_PO_4_ solutions. Uncoated fused-silica capillary 75 μm id × 62.5 cm (71 cm to the detector); hydrodynamic injection using 40 mbar × 4 s; separation voltage at 15 kV; UV detection at 195 nm.

Finally, the effect of CD concentration on separation was evaluated to determine whether smaller amounts of CDs could promote effective separation without significantly compromising *R*_s_ values. It is noteworthy that charged CDs, such as CM-β-CD (which remains anionic across the entire pH range), are commonly used for separating charged enantiomers, as dipole–dipole interactions can facilitate analyte-selective binding, and the opposing mobilities between CDs and enantiomers may further enhance separation efficiency. Nevertheless, excessive CD concentrations can increase current values and compromise the reproducibility of analyses. Figure 4 and Table 2 show that a decrease the concentration of CM-β-CD up to 2.5 mg mL^–1^ resulted in a gradual decrease in *R*_s_. However, the use of 2.5 mg mL^–1^ CD was chosen as the optimal condition, since the baseline separation of enantiomers was obtained in a shorter analysis time, employing a lower concentration (consumption) of CD, thus achieving a good cost-benefit ratio.

### 3.2 Computational Section

To elucidate the molecular basis of EMO, we investigated the conformational preferences and energetics of diastereomeric complexes formed between CM-β-CD and AML enantiomers. The topological features and binding affinities of these complexes were characterized through computational modeling. These insights enabled us to rationalize the observed differences in electrophoretic mobility and to assign specific peaks in the electropherogram to individual enantiomers.

**Figure 5:**
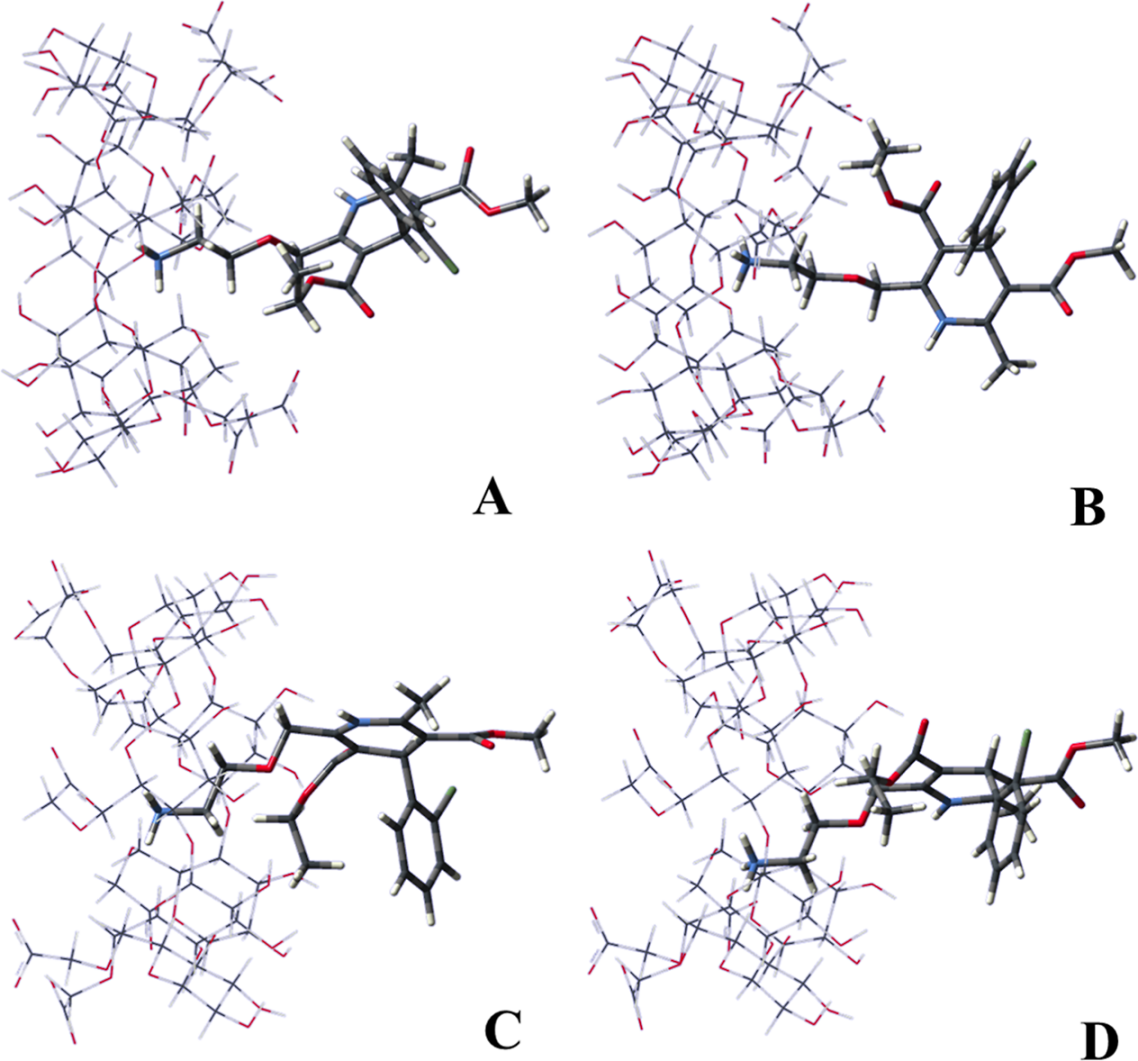
PM3/DFT(B97D/6-31G(d,p)) fully optimized geometries for the complexes: (a) (+)-(*R*)-AML/CM-β-CD (mode 1), (b) (−)-(*S*)-AML/CM-β-CD (mode 1) (c) (+)-(*R*)-AML/CM-β-CD (mode 2) and (d) (−)-(*S*)-AML/CM-β-CD (mode 2).

Table 4 presents the electronic energy (Δ*E*) and Gibbs free energy (Δ*G*) values for the AML@CM-β-CD complexes (Figure 5), calculated in the aqueous phase at the B97D/6-31G(d,p) level. The complexes were named (+)-(*R*)-AML/CM-β-CD (mode 1), (−)-(*S*)-AML/CM-β-CD (mode 1), (+)-(*R*)-AML/CM-β-CD (mode 2) and (−)-(*S*)-AML/CM-β-CD (mode 2), in which the terms “mode 1” and “mode 2″ refer to the inclusion of AML through the smaller cavity (primary face) and larger cavity (secondary face), respectively.

**Table 4:** Electronic Energy (Δ*E*) and Gibbs Free Energy (Δ*G*) Obtained from B97D/6-31(d,p)//PM3 Calculations, in Aqueous Solution (SMD Method), for the Inclusion Complexes Formed by AML Enantiomers and CM-β-CD^a^

complexes	Δ*E*	Δ*G*
(+)-(*R*)-AML/CM-β-CD (mode 1)	–19.4	–3.1
(−)-(*S*)-AML/CM-β-CD (mode 1)	–19.9	–3.2
(+)-(*R*)-AML/CM-β-CD (mode 2)	–24.2	–4.5
(−)-(*S*)-AML/CM-β-CD (mode 2)	–29.6	–6.7

a Values in kcal mol^–1^.

The analysis of (+)-(*R*)-AML/CM-β-CD and (−)-(*S*)-AML/CM-β-CD complexes in mode 1 indicates negligible energetic disparities, as evidenced by their nearly identical Δ*E* and Δ*G* values. This observation suggests that the enantiomers interact with CM-β-CD in a structurally equivalent manner. The limited conformational flexibility of the smaller CM-β-CD cavity likely restricts the optimal positioning of the bulky AML molecules, leading to steric clashes and electrostatic repulsion that mitigate any substantial energetic preference for one enantiomer over the other.

Comparative analysis of (+)-(*R*)-AML/CM-β-CD and (−)-(*S*)-AML/CM-β-CD complexes in mode 2 indicates a marginally enhanced stability for the (−)-(*S*)-AML complex, as evidenced by slightly more favorable Δ*E* and Δ*G* values. This finding suggests a geometric predisposition of the larger CD cavity to accommodate the bulkier AML molecule. The increased cavity diameter enables deeper penetration and more efficient inclusion, thereby fostering stronger intermolecular interactions between the host and guest molecules.

**Figure 6:**
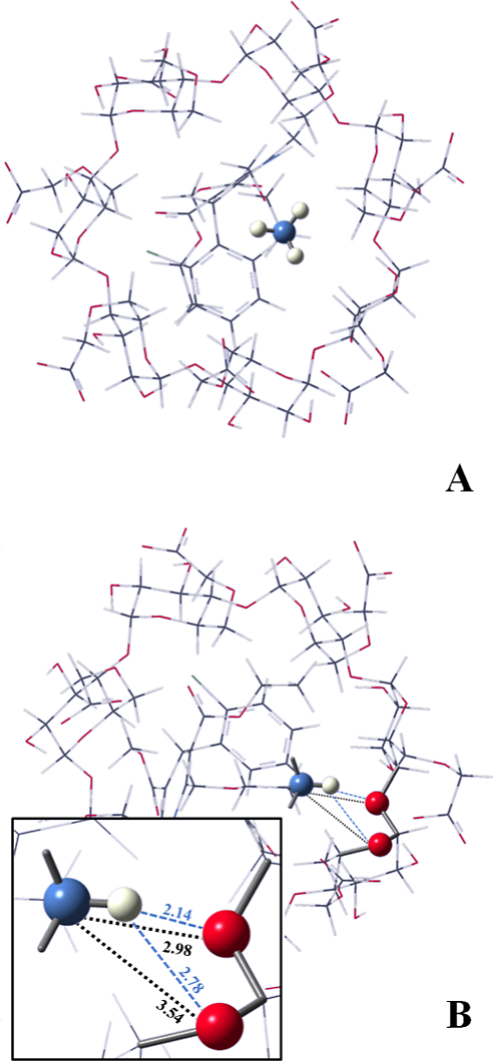
PM3/DFT(B97D/6-31G(d,p)) fully optimized geometries for the complexes: (a) (+)-(*R*)-AML/CM-β-CD (mode 2) and (b) (−)-(*S*)-AML/CM-β-CD (mode 2).

Structural analysis reveals that the (−)-(*S*)-AML enantiomer exhibits a more favorable binding affinity with CM-β-CD in mode 2, as depicted in Figure 6. The enhanced interaction is attributed to the formation of two hydrogen bonds (2.14 and 2.78 Å) between the amino group’s hydrogen atoms (–NH_3_^+^) and the oxygen atoms of CM-β-CD. Moreover, two electrostatic interactions (2.98 and 3.54 Å) arise from the interaction of the amino group’s free electron pair with the oxygen atoms of CM-β-CD. In contrast, the (+)-(*R*)-AML complex lacks such stabilizing interactions. The stronger intermolecular forces present in the (−)-(*S*)-AML/CM-β-CD (mode 2) complex contribute to its greater thermodynamic stability and, consequently, its prolonged *T*_m_ within the electrophoretic system (Figure 6).

## 4 Conclusions

This study has effectively established an efficient methodology for the enantioselective *R*_s_ of AML utilizing CE via the meticulous optimization of pivotal parameters. The influence of both the composition and concentration of the BGE was examined, with 50 mM phosphate BGE identified as the optimal milieu. Furthermore, the impact of BGE pH on the separation was also investigated, with pH 4.0 demonstrating superior outcomes in terms of *R*_s_ and *T*_m_. The use of CM-β-CD facilitated the separation of the AML enantiomers, in which the highest *R*_s_ was obtained using a concentration of 2.5 mg mL^–1^ of chiral selector, archiving an *R*_s_ equal to 1.68. Computational analysis indicates that the disparity between Δ*E* and Δ*G* values provides a reliable metric for predicting EMO in CCE. The (−)-(*S*)-AML/CM-β-CD complex (mode 2) exhibits energetically favorable interactions with the CM-β-CD compared to its (+)-(*R*)-enantiomer (mode 2). These findings underscore the synergistic benefits of computational and experimental approaches in elucidating chiral discrimination mechanisms and identifying EMO in CCE.
